# Editorial: Parenthood and parental wellbeing: exploring diverse trajectories and influences

**DOI:** 10.3389/fsoc.2026.1861760

**Published:** 2026-05-19

**Authors:** Alessandra Decataldo, Sara Martucci, Alessandra Minello, Livia Elisa Ortensi

**Affiliations:** 1 Department of Sociology and Social Research, University of Milano-Bicocca, Milan, Italy; 2 John Jay College, City University of New York, New York, NY, United States; 3 Department of Statistical Sciences, University of Padua, Padua, Italy; 4 Department of Statistical Sciences, University of Bologna, Bologna, Italy

**Keywords:** fertility trajectories, gendered inequalities, parental wellbeing, parenthood, socio-economic inequalities

## 1 Introduction: rethinking parenthood and parental wellbeing today

The transformations that have reshaped parenthood in contemporary societies constitute the starting point of this Research Topic, which seeks to develop an integrated and theoretically grounded understanding of parental wellbeing. Over recent decades, processes such as the pluralization of family forms, the reconfiguration of welfare arrangements and legal frameworks, the diffusion of assisted reproductive technologies, increasing economic uncertainty, and the postponement of fertility have significantly altered the meanings, expectations, and practices associated with becoming and being a parent. Parental wellbeing can no longer be conceptualized as a stable individual attribute; rather, it emerges as a complex, dynamic, and context-dependent outcome shaped by the interplay of multiple dimensions, including physical and mental health, caregiving responsibilities, cultural expectations, institutional arrangements, and work-related constraints (Nomaguchi and Milkie, 2020).

The contributions collected here converge in demonstrating that wellbeing is produced through the interaction between distinct yet interdependent domains. First, reproductive trajectories and desires play a crucial role in shaping expectations, identities, and emotional outcomes (Greil et al., 2010).

Second, institutional infrastructures provide the material and symbolic resources through which parenting is enacted, thereby structuring opportunities for reconciliation between work and family life (Esping-Andersen, 2009).

Third, gender regimes and moral frameworks continue to exert a powerful influence on parental wellbeing by defining normative expectations of “good” parenting. The persistence of ideologies such as intensive mothering contributes to gendered inequalities in both caregiving and wellbeing (Hays, 1996).

Finally, work environments and increasingly pervasive digital contexts represent key arenas in which parental roles are negotiated and experienced (O'Brien et al., 2025), influencing both the distribution of time and resources and the construction of parental identities.

The articles in this Research Topic advance a conceptualization of parental wellbeing as an emergent property of situated interactions across life-course trajectories, institutional contexts, and normative orders. By moving beyond individualistic and static approaches, they highlight the need to account for the layered and relational nature of wellbeing, as well as for the structural inequalities and cultural expectations that shape it.

## 2 Reading the contributions together: a four-axis analytical framework

The numerous stressors of parenting, including, but not limited to, reproductive care, finances, child care arrangements, postpartum care, daily logistics and the physical and emotional tolls of childrearing are most frequently felt individually or within the immediate family. While the articles in this Research Topic differ on their emphases, the overarching directive is the need to examine these issues at structural levels including workplace accommodations and national and local policies around care. Four overlapping themes emerge across the 11 papers included in this Research Topic. These themes highlight the globally shared challenges around reproduction and parenting, the persistence of long-term societal and interpersonal issues, and ways of coping with these issues via digital communities.

The role of digital and online communities as supportive spaces emerges clearly in the study by Decataldo and Andreoni, which examines how individuals undergoing assisted reproductive technology (ART) navigate reproductive desire and stigma through participation in online environments. The digital mediation of pregnancy is further explored by Picardi and Agodi, who analyze online discourses surrounding 3D and 4D fetal ultrasound images. A related perspective is offered by Falzea, who investigates how Italian fathers discuss masculinity, care, and parental wellbeing in an online forum.

The gendered organization of care and parental responsibility is addressed by Vettoretto et al., who examine the mental labor carried by Italian mothers. Attention to more complex and demanding parenting contexts is provided by Simmat-Durand and Toutain, who explore the experiences of families raising children affected by prenatal alcohol exposure.

A broader perspective about structural and institutional influences on parenting is offered by Tartakovsky and Mizrahi, who investigate the motivations underlying childbearing intentions and their relationship with personal values and socio-demographic characteristics. This perspective is complemented by Maruya et al., whose analysis of survey data from Japan reveals increasing openness among younger generations to shared caregiving models. Similarly, Wang et al. demonstrate how the expansion of childcare provision in China is positively associated with fertility intentions. At the discursive and policy level, Bandelli analyzes the “First 1,000 Days” framework in Italy.

Finally, the emotional wellbeing in reproduction and parenthood is further explored in healthcare and workplace contexts. Decataldo et al. investigate communication dynamics between healthcare professionals and parents in Neonatal Intensive Care Units. In parallel, Hadley et al. introduce the concept of *reproductive capital* to examine how workplace environments influence experiences of fertility, infertility, and assisted reproduction.

## 3 Reframing parental wellbeing: cross-cutting insights

Taken together, the contributions in this Research Topic converge on a set of cross-cutting findings that help reframe how parental wellbeing is conceptualized and studied.

First, parental wellbeing emerges as a multi-situated condition, shaped across different spaces and transitions. It unfolds across the home, the workplace, healthcare settings, and digital environments, and evolves along the life course. Wellbeing is not a static outcome but a process, continuously negotiated as individuals move across institutional contexts and relational settings.

Second, gender inequalities operate as a recurring and structuring mechanism, although they take heterogeneous forms across contexts. The gendered division of care remains visible in the unequal distribution of domestic labor, but it also extends into less visible domains. These inequalities appear as patterned dynamics that shape both experiences and perceptions of wellbeing.

Third, institutions matter. On one hand, they act as infrastructures that shape the conditions under which parenthood is experienced. On the other, they function as relational arenas in which interactions with professionals and organizations can generate either support or stress.

Fourth, norms and public discourses emerge as powerful, albeit often implicit, regulatory forces. Across these dimensions, the contributions collectively challenge the idea of a single, linear transition to parenthood. Instead, they point to a plurality of trajectories through which individuals encounter and navigate parenthood, with unequal distributions of risks, stigma, and resources.

This Research Topic highlights the need for analytical approaches that move beyond individual-level explanations and account for the interplay between life-course trajectories, institutional arrangements, and normative frameworks.
